# Energy-Efficient Collaborative Task Computation Offloading in Cloud-Assisted Edge Computing for IoT Sensors

**DOI:** 10.3390/s19051105

**Published:** 2019-03-04

**Authors:** Fagui Liu, Zhenxi Huang, Liangming Wang

**Affiliations:** 1 School of Computer Science and Engineering, South China University of Technology, Guangzhou 510006, China; fgliu@scut.edu.cn; 2 School of Software Engineering, South China University of Technology, Guangzhou 510006, China

**Keywords:** edge computing, computation offloading, collaborative task, energy efficiency, Internet of Things

## Abstract

As an emerging and promising computing paradigm in the Internet of things (IoT), edge computing can significantly reduce energy consumption and enhance computation capability for resource-constrained IoT devices. Computation offloading has recently received considerable attention in edge computing. Many existing studies have investigated the computation offloading problem with independent computing tasks. However, due to the inter-task dependency in various devices that commonly happens in IoT systems, achieving energy-efficient computation offloading decisions remains a challengeable problem. In this paper, a cloud-assisted edge computing framework with a three-tier network in an IoT environment is introduced. In this framework, we first formulated an energy consumption minimization problem as a mixed integer programming problem considering two constraints, the task-dependency requirement and the completion time deadline of the IoT service. To address this problem, we then proposed an Energy-efficient Collaborative Task Computation Offloading (ECTCO) algorithm based on a semidefinite relaxation and stochastic mapping approach to obtain strategies of tasks computation offloading for IoT sensors. Simulation results demonstrated that the cloud-assisted edge computing framework was feasible and the proposed ECTCO algorithm could effectively reduce the energy cost of IoT sensors.

## 1. Introduction

With the explosive development of the Internet of Things (IoT), enormous sensors are connected through IoT techniques, and these sensors generate massive amounts of data and demand [1,2,3,4]. However, due to the limitations of size, battery life and heat dissipation in IoT sensors, severely constrained resources cannot meet the increasingly complex application requirements [5]. Since the cloud has adequate computation resources, cloud computing [6] has been proposed as an effective way to tackle the above challenges. By offloading computation tasks to cloud data centers, cloud computing can extend the computation power of IoT sensors.

However, cloud data centers are mostly far from IoT sensors, which causes significant communication overhead and severely lessens the offloading efficiency. They are usually unacceptable for some latency-sensitive IoT applications. Thus, edge computing [7,8], e.g., multi-access edge computing (MEC) [9] and fog computing [10,11], as a complement and extension of the cloud computing paradigm, is a prospective solution that can overcome the aforementioned challenges. In edge computing, computation resources are deployed near devices, such as smart gateways, access points, base stations, etc., and integrated as edge servers. The resource-constrained device can offload the computing task to the edge server via single-hop wireless transmission. The edge server then performs the computation and returns the computation result. Different from cloud computing, edge computing can provide computing resources at the network edge. Therefore, it can reduce communication latency and network bandwidth requirement [12,13]. Furthermore, based on the advantages of edge computing, many efforts have explored its potential in practical applications, such as video analysis [14], fault detection [15] and vehicular networks [16].

Computation offloading [17,18,19] is a key technique of edge computing to efficiently enhance the computation capability of IoT sensors. In addition, computation offloading can save computation energy consumption of IoT sensors at the cost of additional transmission energy consumption. Therefore, balancing the tradeoff between computation and communication costs in order to optimize offloading strategies is a key challenge of computation offloading problem. Many previous studies on computation offloading in the field of edge computing have been proposed [20]. Most of the literature optimize the offloading strategies under certain constraints, such as task completion deadline or bandwidth resource constraints, to achieve system performance gains, like reducing energy consumption or latency. To improve the system efficiency, Dinh et al. [21] observed performance gain in energy and latency when offloading decisions, task assignment, and CPU frequency of the device were jointly considered. Ref. [22] jointed optimization of the computation offloading decisions and the allocation of computation resources, transmission power, and radio bandwidth in a hybrid fog/cloud system. However, most works assume that computing tasks are independent. That is, computation offloading with inter-task dependency relationships, especially the task dependency among various devices, have seldom been considered and addressed. This kind of inter-task dependency is ubiquitous in the IoT environment such as smart home [23], smart healthcare [24], and smart city [25,26,27]. For example, consider a scenario where multiple IoT sensors are combined to complete an IoT service. One of the sensors needs to combine the data processed by other sensors for calculation. There is data dependency between these IoT sensors, meaning that different tasks between IoT sensors need to exchange data to obtain the expected results. In general, making computation offloading strategies in the restriction of task dependency relationships is a challenging problem.

In this paper, to tackle the inter-task dependency problem mentioned above, we modeled the task computation offloading problem of IoT sensors as an energy optimization problem while satisfying inter-task dependency and service completion time constraint. Particularly, these tasks with dependency among various sensors were referred to as the collaborative task. Compared to the cloud data center, the computation capability and resources of the edge server are limited. Therefore, for the network architecture, similar to some previous works [22,28,29], we described a cloud-assisted edge computing framework as a three-tier network architecture, which consisted of IoT sensors, an edge computing server, and a remote cloud server. The computing task of the IoT sensor could be performed locally, offloaded to the edge server, or further forwarded to the cloud server. The main contributions of this paper are summarized as follows:

- Taking inter-task dependency and service completion time constraint into consideration, we formulated the computation offloading strategy problem as a mixed integer optimization problem on the cloud-assisted edge computing framework, aimed at minimizing the energy consumption of IoT sensors. Since the problem is a NP-hard problem, solving such problems is challenging.
- Based on convex optimization theory, we proposed an Energy-efficient Collaborative Task Computation Offloading (ECTCO) algorithm to solve the optimization problem. The algorithm obtains computation offloading decisions through a semidefinite relaxation (SDR) [30] approach and probability-based stochastic mapping method.
- We performed extensive simulations to evaluate the proposed method. Simulation results showed that in the inter-task dependency scenario, the proposed ECTCO algorithm outperformed in terms of energy consumption compared to existing algorithms in computation offloading. Moreover, the performance evaluations verified the effectiveness and the adaptability of the proposed algorithm under different system parameters.

The remainder of this paper is organized as follows. Related works are reviewed in Section 2. Section 3 introduces the system model and formulates an optimization problem. In Section 4, we present the SDR approach to solve the optimization problem and propose the ECTCO algorithm. Simulation results are presented and discussed in Section 5. Finally, Section 6 draws conclusions and discusses future work.

Notation: in this paper, the mathematical symbols follow the rules as follows. The italic letter denotes a variable, and the uppercase letter with calligraphic font denotes a set. The bold lowercase letter denotes a vector, while the bold uppercase letter denotes a matrix. $g^{T}$ and $G^{T}$ represent the transpose of vector g and matrix G, respectively. The trace function of matrix G is denoted by Tr(G).

## 2. Related Works

Computation offloading is an attractive and challenging topic in edge computing. It has been extensively investigated with a variety of architectures and offloading schemes. Generally speaking, task computation offloading can be classified into two computing models [17]: Binary offloading  [28,29,31,32] and partial offloading [33,34,35].

For binary offloading, the computation task is either executed locally or offloaded as a whole. We further divide the relevant researches into a two-tier network [31,32] and a three-tier network [22,28,29]. In [31], You et al., discussed the resource allocation problem based on TDMA and OFDMA in multi-user computation offloading system. The computation offloading strategy was obtained via the dynamic channel conditions. The results showed that OFDMA access enables higher energy savings compared to the TDMA system. Taking the scenario of edge caching into consideration, Hao et al. [32] jointly optimized a task offloading and cache problem to improve energy efficiency. All of the studies above focus on a two-tier network consisting of devices and edge nodes only. In a three-tier network, the optimization problem of computation offloading strategy becomes more complicated. To achieve a higher energy efficiency, Ma et al. [28] devised a distributed computation offloading algorithm in the cloud-edge interoperation system by utilizing game theory. Zhao et al. [29] proposed a QoS guaranteed offloading policy by coordinating the edge cloud and the remote cloud under the delay bounded.

For partial offloading, the computation task is segmented into a set of sub-tasks. Some of the sub-tasks can be executed locally, and the rest are offloaded to the edge. In [33], Wang et al. combined the dynamic voltage scaling technique with partial computation offloading and proposed a local optimal algorithm by using the univariate search technique to achieve the goal of reducing energy consumption and shortening the delay. In [34], the authors integrated wireless power transfer (WPT) technology into the edge computing system to power the multi-user computation offloading. Ren et al. [35] presented a novel partial computation offloading model to optimize the weighted-sum latency-minimization resource allocation problem of multi-user edge computing system. However, the aforementioned studies about computation offloading in edge computing including binary offloading and partial offloading do not consider the important inter-task dependency among various devices.

Recently, there have been some works on computation offloading with task dependency in the field of cloud computing [36,37]. In the single-user case, Zhang et al. [36] modeled an application as general topology, and proposed the one-climb policy and Lagrange relaxation method to resolve the delay-constrained workflow scheduling problem. In [37], Guo et al. investigated a multi-user scenario in the cloud computing environment, where each individual device had an application that could be partitioned into multiple sub-tasks with dependency, with the goal of optimizing the energy efficiency of the computation offloading. However, both of them divided a complex application into multiple sub-tasks by an individual device, taking into account the dependency between them. In contrast, we considered the inter-task dependency suitable to IoT scenario, that is, the inter-task dependency among IoT sensors. These tasks were simple computing tasks that can be fully offloading. Furthermore, different from the cloud computing field mentioned above, in this paper, we focused on cloud-assisted edge computing framework of the three-tier network architecture, in which the computation offloading decision considering the inter-task dependency was much more complicated.

## 3. System Model and Problem Formulation

In this section, we describe the computation offloading in IoT scenario for collaborative task. Then the system model is introduced, followed by the communication, computation and task dependency model. Finally, the optimization problem of collaborative task computation offloading is formulated. The main symbols and parameters throughout this paper are summarized in Table 1.

### 3.1. Scenario Description

**Figure 1:**
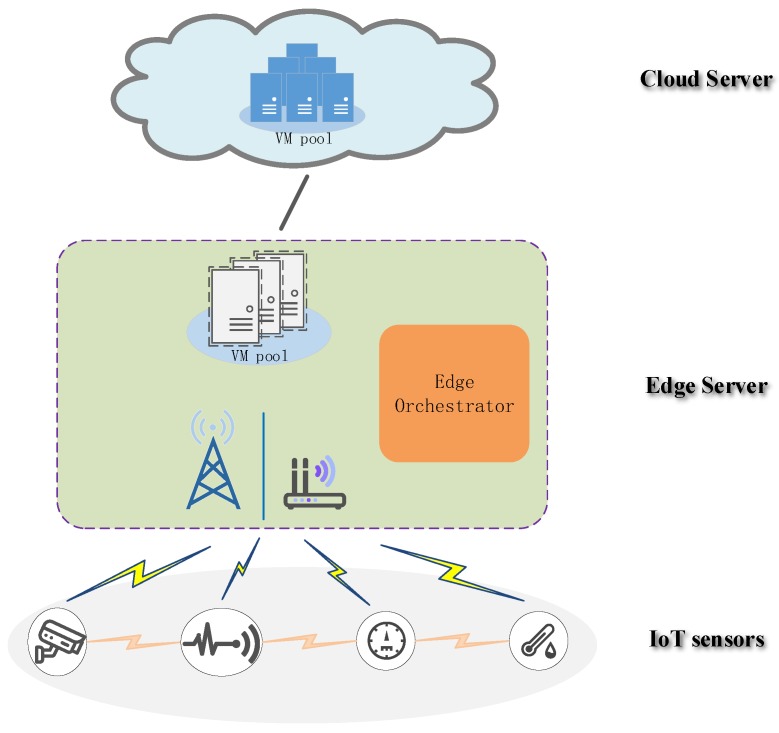
Cloud-assisted edge computing framework with collaborative task.

We considered an IoT service *S* in the system that required *K* IoT sensors for collaborative computing. The IoT service was modeled as *K* fine-grained computing tasks distributed among *K* different sensors. There was data dependency among the computing tasks of different sensors. As shown in Figure 1, a three-tier network architecture consisting of *K* IoT sensors, one edge server, and a remote cloud server was presented. Each sensor had a computation task to be handled. We denoted the set of tasks as K≜{1,2,…,K}, which were preemptive and indivisible work unit. Direct communication was created between sensors via the wireless link (e.g., M2M and D2D communication) to exchange task calculation results with relevant dependent tasks. Each sensor was connected to the edge server via a wireless link (e.g., 5G and WiFi), while the edge server was connected to the remote cloud through a wired link such as fiber.

In this paper, the edge orchestrator at the edge server performed as a computation offloading management module, which decided whether the computing task was executed locally, offloaded to the edge server, or forwarded to the cloud server through the edge server. Similar to many existing studies [22,37,38], we considered our model and proposed method in a quasi-static scenario where all IoT sensors remained unchanged during a computation offloading period (usually within hundreds of milliseconds or several seconds). The computation resources of the edge server and the remote cloud server were represented by the virtual machine (VM), each of which had fixed computation capability.

For IoT service *S*, to satisfy QoS, we denoted $T_{s}^{max}$ as the completion deadline for service *S*. For heterogeneous computing tasks in the service, we defined task attribute $U_{k}≜\left\{\omega_{k},d_{k}\right\}$, where $d_{k}$ (in bits) was the data size of computation task *k*, and $\omega_{k}$ (in CPU cycles/bit) was the amount of computation resources required for each bit in task *k*, which depended on the computational complexity of the computation task [33]. In addition, we denoted $C_{k}$ as the size of computation resources amount needed to complete task *k*, and $C_{k}=\omega_{k}d_{k}$. We assumed that $T_{s}^{max}$ and $U_{k}$ were known before the task offloading and would not change during the offloading period.

### 3.2. Communication Model

We first introduced the communication model and gave the uplink data rate when the IoT sensor offloaded the computation task to the edge server. We denoted $p_{k}^{tra}$ as the transmission power of task *k*. We let $H_{k}$ be the channel gain between the sensor and the edge when transmitting task *k* due to path loss and shadowing attenuation. According to the Shannon formula, the uplink transmission rate for task *k* could be given by

(1) $$r_{k}=Blog_{2}\left(1+\frac{p_{k}^{tra}H_{k}}{\sigma^{2}}\right),$$

where *B* is channel bandwidth and $\sigma^{2}$ denotes the variance of complex white Gaussian channel noise.

In this paper, similar to many previous works on edge computing [22,29,31], we ignored the transmission delay of task output. This was because the data size after task computing was generally small compared to task input, usually only hundredth or thousandth part of task input. For example, the size of task output was a few KB while the size of task input was hundreds of KB or a few MB. Therefore, to extract some insights, only the uplink transmission rate between the IoT sensor and the edge server was considered.

### 3.3. Computation Model

#### 3.3.1. Local Computing

For local task computing, let $f_{k}^{l}$ be the computation capability of task *k* on the sensor. Thus, the computation execution time of task *k* by local computing could be expressed as

(2) $$T_{k}^{loc}=\frac{C_{k}}{f_{k}^{l}}.$$

The energy consumption per computing cycle was defined as $\varepsilon =\kappa f^{2}$, where κ was the effective switched capacitance depending on the chip architecture [39]. Then the corresponding energy consumption for local computing could be computed as

(3) $$E_{k}^{loc}=\kappa {\left(f_{k}^{l}\right)}^{2}C_{k}.$$

#### 3.3.2. Edge Computing

For task computing on the edge server, the processing of task *k* included two phases in sequence: (i) Transmitting phase, the IoT sensor transmitted the data of task *k* to the edge via wireless transmission (ii) edge computing phase, task *k* was executed in the edge. Therefore, the delay of the edge processing task was the sum of the wireless link transmission delay and the edge server computing delay. The total delay and energy consumption of edge computing for task *k* were given respectively by

(4) $$T_{k}^{edge}=\frac{d_{k}}{r_{k}}+\frac{C_{k}}{f_{k}^{e}},$$

(5) $$E_{k}^{edge}=p_{k}^{tra}\left(\frac{d_{k}}{r_{k}}\right)+p_{k}^{cir}\left(\frac{C_{k}}{f_{k}^{e}}\right),$$

where $f_{k}^{e}$ indicates the computation resources of the edge allocated to task *k*, and $p_{k}^{cir}$ is the constant idle circuit power (e.g., the digital-to-analog converter (DAC)) when the IoT sensor is idle.

#### 3.3.3. Cloud Computing

If a computing task was offloaded to the cloud server, the IoT sensor first transmitted the data of the task through wireless transmission to the edge, and then the edge server forwarded the data to the cloud via the wired link. Thus, the latency of the cloud processing task was the sum of the wireless link transmission delay, the wired link transmission delay, and the cloud server computing delay. We denoted $R_{k}^{EC}$ as the rate of the wired link allocated to task *k* transmission between the edge and the cloud. The computation resources of the cloud assigned to task *k* were $f_{k}^{c}$. Then the delay and energy consumption of cloud computing were written respectively as

(6) $$T_{k}^{cloud}=\frac{d_{k}}{r_{k}}+\frac{d_{k}}{R_{k}^{EC}}+\frac{C_{k}}{f_{k}^{c}},$$

(7) $$E_{k}^{cloud}=p_{k}^{tra}\left(\frac{d_{k}}{r_{k}}\right)+p_{k}^{cir}\left(\frac{d_{k}}{R_{k}^{EC}}+\frac{C_{k}}{f_{k}^{c}}\right).$$

### 3.4. Task Dependency Model

**Figure 2:**
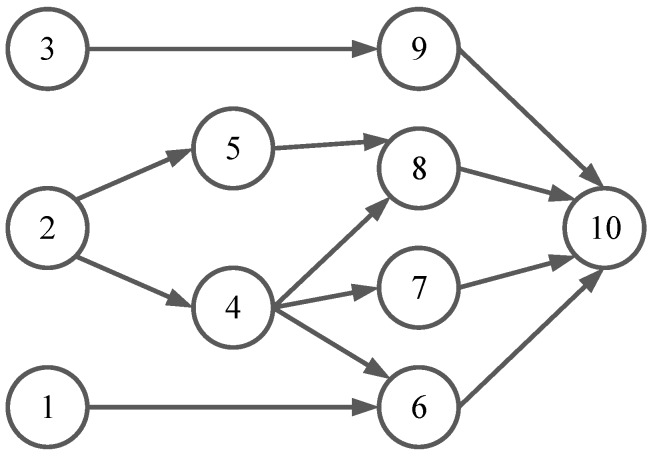
An example of dependency relationships among tasks.

To model the data dependency relationships of computing tasks among IoT sensors, we utilized a directed acyclic graph $G_{s}=\left(V,A\right)$, where *V* denoted the node set for computing tasks, and each node *i* in $G_{s}$ represented a computing task. The dependency relationship among tasks was represented by the directed arc set in set *A*. A directed arc a(i,j) in set *A* indicated the precedence constraint between adjacent task *i* and task *j*, which meant that task *j* could not start execution until its precedent task *i* was completed. In addition, the node without predecessors was defined as starting node, and the node without descendant was the ending node. There could be multiple starting nodes, which could perform computing tasks in parallel, while only one ending node, indicating the completion node of the IoT service. Computing task on each sensor can be executed on the local, edge, or cloud. An example of dependency relationships among 10 tasks is shown in Figure 2. The immediate predecessors of task 8 were task 5 and task 4, and its descendant was task 10. The starting nodes were tasks 1, 2, and 3, while task 10 was the ending node.

The data dependency among tasks affected the computation offloading strategy through the completion time of the task. To consider these dependency relationships in the task offloading model, we gave the definition of finish time and ready time of a computing task.

**Definition** **1** (Finish Time)**.** *The finish time of a task is defined as the time at which the task has fully completed execution. Thus, the finish time of task k, denoted by ${FT}_{k}$ is given by*

(8) $${FT}_{k}=RT_{k}+T_{k},$$

*where ${RT}_{k}$ is the ready time of task k and $T_{k}$ denotes the execution time of task k.*

**Definition** **2** (Ready Time)**.** *The ready time of a task is defined as the earliest start time when all its immediate predecessor tasks have completed. Thus, the ready time of task k, denoted by ${RT}_{k}$ can be expressed as*

(9) $$RT_{k}=\left\{\begin{array}{cc} \underset{j\in P(k)}{max}{FT}_{j}, & P(k)\neq ⌀,\\ 0, & P(k)=⌀, \end{array}\right.$$

*where P(k) denotes the set of the immediate predecessor tasks of task k.*

We can observe from Equation (9) that when P(k) was empty, task *k* had no immediate predecessor, which meant task *k* was the starting node and ${RT}_{k}$ was equal to zero. It was assumed that the transmission time of the task calculation result was negligible, so it could be considered that the maximum finish time in immediate predecessors of task *k* was the ready time of task *k*.

### 3.5. Problem Formulation

We denoted the offloading strategies for task *k* as $x_{k},y_{k},z_{k}\in \left\{0,1\right\}$, meaning task *k* was executed locally, at the edge, or at the cloud, respectively. The offloading placement decisions satisfied the following constraint

(10) $$x_{k}+y_{k}+z_{k}=1,\forall k\in K,$$

where only one of the three variables for task *k* could be 1.

According to Equations (2)–(7) and (10), the execution time and the executing energy consumption of task *k* could be expressed respectively as

(11) $$T_{k}=T_{k}^{loc}x_{k}+T_{k}^{edge}y_{k}+T_{k}^{cloud}z_{k},$$

(12) $$E_{k}^{exec}=E_{k}^{loc}x_{k}+E_{k}^{edge}y_{k}+E_{k}^{cloud}z_{k}.$$

Due to the data dependency among tasks, task *k* needed to wait for its predecessors to complete before executing. Thus, the energy consumption during the waiting period of task *k*, called waiting energy consumption, was defined as

(13) $$E_{k}^{wait}=p_{k}^{cir}RT_{k}.$$

According to Equations (12) and (13), the total energy consumption of computing task *k* was

(14) $$E_{k}=E_{k}^{exec}+E_{k}^{wait}.$$

Based on the above models, we proposed to minimize the energy consumption of all sensors in the system by jointly optimizing the task offloading strategy and the task ready time. The task offloading strategy was formulated as $\gamma =[x_{1},y_{1},z_{1},\ldots ,x_{K},y_{K},z_{K}]$ and the ready time was $R=[{RT}_{1},{RT}_{2},\ldots ,{RT}_{K}]$. Therefore, the optimization problem of minimizing the energy consumption could be modeled as follows:

(15) $$\begin{array}{ll} & \underset{\gamma ,R}{\mathrm{minimize}}\sum_{k=1}^{K}E_{k}\\ subject\;to\; & C1:x_{k},y_{k},z_{k}\in \left\{0,1\right\},\forall k\in K,\\ & C2:x_{k}+y_{k}+z_{k}=1,\forall k\in K,\\ & C3:{FT}_{K}⩽T_{s}^{max},\\ & C4:{RT}_{k}=\max_{j\in P(k)}{FT}_{j},P\left(k\right)\neq ⌀,\forall k\in K,\\ & C5:RT_{k}=0,P\left(k\right)=⌀,\forall k\in K, \end{array}$$

where *C*1 and *C*2 are the constraints on the offloading strategy of each task; constraint *C*3 indicates the completion time of the task *K* in ending node was within the maximum tolerable delay of the IoT service *S*; the task precedence constraints *C*4 and *C*5, representing that task *k* started to execute only after all its precedent tasks finish. And tasks executed in parallel at the start of offloading if these tasks were in starting nodes. Due to the binary constraint *C*1, the optimization problem was a mixed integer programming problem, which is a non-convex and NP-hard problem [40].

## 4. Computation Offloading Optimization with Inter-Task Dependency

In this section, to find an effective solution for the optimization problem Equation (15), we first convert it equivalently into a quadratically constrained quadratic programming (QCQP) problem. Then it is transformed into a standard convex problem through SDR approach, and a stochastic mapping method based on offloading probability is proposed to recover the offloading strategy.

### 4.1. QCQP Transformation and Semidefinite Relaxation

Firstly, we replaced the binary constraint *C*1 with a quadratic constraint by

(16) $$x_{k}\left(x_{k}-1\right)=0,y_{k}\left(y_{k}-1\right)=0,z_{k}\left(z_{k}-1\right)=0,\forall k\in K.$$

Then, to transform the problem Equation (15) into a standard QCQP problem, constraint C4 could be rewritten in a linear form as

(17) $${RT}_{k}-FT_{j}\geq 0,\forall j\in P\left(k\right),k\in K.$$

Now the optimization problem Equation (15) could be equivalently transformed as

(18) $$\begin{array}{ll} & \underset{\gamma ,R}{\mathrm{minimize}}\sum_{k=1}^{K}E_{k}\\ subject\;to\; & x_{k}\left(x_{k}-1\right)=0,y_{k}\left(y_{k}-1\right)=0,z_{k}\left(z_{k}-1\right)=0,\forall k\in K,\\ & x_{k}+y_{k}+z_{k}=1,\forall k\in K,\\ & {FT}_{K}⩽T_{s}^{max},\\ & {RT}_{k}-FT_{j}\geq 0,\forall j\in P\left(k\right),k\in K,\\ & RT_{k}=0,P\left(k\right)=⌀,\forall k\in K. \end{array}$$

Next, we vectorized the variables into a vector with size of (4K+1)×1, denote $q={\left[\gamma ,R,1\right]}^{T}$. Define $e_{j}$ and $e_{j}^{\prime }$ as standard unit vectors of (4K+1)×1 and 4K×1, respectively, and their *j*th entry was 1. In addition, diag($e_{j}$) was the diagonal matrix, of which diagonal elements were the elements of vector $e_{j}$. The optimization problem Equation (18) could now be converted into the following standard QCQP formulation

(19) $$\begin{array}{ll} & \underset{q}{\mathrm{minimize}}\;{\left(b_{0}\right)}^{T}q\\ subject\;to\; & q^{T}\mathrm{diag}\left(e_{j}\right)q-{\left(e_{j}\right)}^{T}q=0,j=1,\ldots ,3K,\\ & {\left(b_{k}^{P}\right)}^{T}q=1,\forall k\in K,\\ & {\left(b_{1}\right)}^{T}q⩽T_{s}^{max},\\ & {\left(e_{3K+k}^{{}^{\prime }}\right)}^{T}q-{\left(b_{2}\right)}^{T}\mathrm{diag}\left(b_{j}^{I}\right)q\geq 0,\forall j\in P\left(k\right),k\in K,\\ & {\left(e_{3K+k}\right)}^{T}q=0,P\left(k\right)=⌀,\forall k\in K, \end{array}$$

where$b_{0}={\left[E_{1}^{loc},E_{1}^{edge},E_{1}^{cloud},\ldots E_{K}^{loc},E_{K}^{edge},E_{K}^{cloud},p_{1}^{cir},p_{2}^{cir},\ldots ,p_{K}^{cir},0\right]}^{T}$,
$b_{k}^{P}=e_{3k-2}+e_{3k-1}+e_{3k}$,
$b_{1}={\left[0_{1\times (3K-3)},T_{K}^{loc},T_{K}^{edge},T_{K}^{cloud},0_{1\times (K-1)},1,0\right]}^{T}$,
$b_{2}={\left[T_{1}^{loc},T_{1}^{edge},T_{1}^{cloud},\ldots T_{K}^{loc},T_{K}^{edge},T_{K}^{cloud},1_{1\times K}\right]}^{T}$,
$b_{j}^{I}=e_{3j-2}^{{}^{\prime }}+e_{3j-1}^{{}^{\prime }}+e_{3j}^{{}^{\prime }}+e_{3K+j}^{{}^{\prime }}.$

By defining $g={\left[q^{T}\;1\right]}^{T}$, the problem Equation (19) could be further transformed into the following equivalent homogeneous QCQP problem

(20) $$\begin{array}{ll} & \underset{g}{\mathrm{minimize}}\;g^{T}M_{0}g\\ subject\;to\; & C6:g^{T}M_{j}g=0,j=1,\ldots ,3K,\\ & C7:g^{T}M_{k}^{P}g=1,\forall k\in K,\\ & C8:g^{T}M_{K}^{D}g⩽T_{s}^{max},\\ & C9:g^{T}M_{kj}^{R}g\geq 0,\forall j\in P\left(k\right),k\in K,\\ & C10:g^{T}M_{k}^{R}g=0,P\left(k\right)=⌀,\forall k\in K, \end{array}$$

where$M_{0}=\left[\begin{array}{cc} 0_{\left(4K+1)\times (4K+1\right)} & \frac{1}{2}b_{0}\\ \frac{1}{2}{\left(b_{0}\right)}^{T} & 0 \end{array}\right]$,
$M_{j}=\left[\begin{array}{cc} \mathrm{diag}(e_{j}) & -\frac{1}{2}e_{j}\\ -\frac{1}{2}{\left(e_{j}\right)}^{T} & 0 \end{array}\right]$,
$M_{k}^{P}=\left[\begin{array}{cc} 0_{\left(4K+1)\times (4K+1\right)} & \frac{1}{2}b_{k}^{P}\\ \frac{1}{2}{\left(b_{k}^{P}\right)}^{T} & 0 \end{array}\right]$,
$M_{K}^{D}=\left[\begin{array}{cc} 0_{\left(4K+1)\times (4K+1\right)} & \frac{1}{2}b_{1}\\ \frac{1}{2}{\left(b_{1}\right)}^{T} & 0 \end{array}\right]$,
$M_{k}^{R}=\left[\begin{array}{cc} 0_{\left(4K+1)\times (4K+1\right)} & \frac{1}{2}e_{3K+k}\\ \frac{1}{2}{\left(e_{3K+k}\right)}^{T} & 0 \end{array}\right]$,
$M_{kj}^{R}=\left[\begin{array}{ccc} 0_{4K\times 4K} & -\frac{1}{2}{\left[{\left(b_{2}\right)}^{T}\mathrm{diag}\left(b_{j}^{I}\right)\right]}^{T} & \frac{1}{2}e_{3K+k}\\ -\frac{1}{2}{\left(b_{2}\right)}^{T}\mathrm{diag}\left(b_{j}^{I}\right) & 0 & 0\\ \frac{1}{2}{\left(e_{3K+k}\right)}^{T} & 0 & 0 \end{array}\right].$

Compared to the optimization problem Equation (15), all constraints had corresponding matrix representations in the optimization problem Equation (20). Particularly, constraint *C*6 corresponded to the integer constraint *C*1, constraint *C*7 was the offloading placement constraint *C*2 while constraint *C*8 was the delay constraint *C*3, and constraints *C*9 and *C*10 came from the task precedence constraints *C*4 and *C*5.

It is worth noting that homogeneous QCQP problem Equation (20) was still a non-convex and NP-hard problem. To solve this problem, we adopted the SDR approach to relax the problem into a semidefinite programming (SDP) problem [30]. Since all vectors were real and all matrices were real symmetric in the problem Equation (20), the SDR conditions were satisfied. We defined additional auxiliary variables $G≜gg^{T}$, which was a rank one symmetric positive-semidefinite matrix. Thus, we had

(21) $$g^{T}M_{0}g=\mathrm{Tr}\left(M_{0}G\right),$$

with rank(G)=1. Then we obtained an equivalent formulation of the optimization problem Equation (20) as follows:

(22) $$\begin{array}{ll} & \underset{G}{\mathrm{minimize}}\;\mathrm{Tr}\left(M_{0}G\right)\\ subject\;to\; & C11:\mathrm{Tr}(M_{j}G)=0,j=1,\ldots ,3K,\\ & C12:\mathrm{Tr}(M_{k}^{P}G)=1,\forall k\in K,\\ & C13:\mathrm{Tr}\left(M_{K}^{D}G\right)⩽T_{s}^{max},\\ & C14:\mathrm{Tr}\left(M_{kj}^{R}G\right)\geq 0,\forall j\in P\left(k\right),k\in K,\\ & C15:\mathrm{Tr}\left(M_{k}^{R}G\right)=0,P\left(k\right)=⌀,\forall k\in K,\\ & C16:G(4K+1,4K+1)=1,\\ & C17:G(4K+1,4K+2)=1,\\ & C18:G(4K+2,4K+1)=1,\\ & C19:G(4K+2,4K+2)=1,\\ & C20:G⪰0,\\ & C21:\mathrm{rank}(G)=1. \end{array}$$

where G⪰0 indicates that matrix G is a positive-semidefinite matrix. In the optimization problem Equation (22), only the rank constraint *C*21 was non-convex, whereas the remaining objective function and constraints were convex. By dropping the rank constraint *C*21, the problem Equation (22) was relaxed to an SDP problem, which could be efficiently solved in polynomial time via using a standard convex optimization solver, such as SeDuMi [41].

### 4.2. Energy-Efficient Collaborative Task Computation Offloading Algorithm (ECTCO)

In this section, due to the inter-task dependency constraint and the service completion time constraint, we improved the random mapping method proposed in [21,42] to obtain the offloading strategy $\gamma^{*}$. Then, a detailed description of the ECTCO algorithm was introduced and the complexity analysis was performed.

We denoted $G^{*}$ as the optimal solution of the optimization problem Equation (22) without the rank one constraint. If the rank of $G^{*}$ was 1, then we could extract the optimal solution of the original problem Equation (15) directly by $G^{*}$. Since $G=gg^{T}$ and g(4K+2)=1, we observed that the last column of G satisfied the following equation:

(23) G(j,4K+2)=g(j),j=1,…,4K+2.

Here, we could use the value of $G^{*}\left(j,4K+2\right)$ to recover the offloading strategy $\gamma^{*}$, for j=1,…,3K.

If $G^{*}$ was not of rank 1, we proposed a stochastic mapping method based on offloading probability to construct a feasible solution of the optimization problem Equation (15). Firstly, we extracted the first 3K elements of the last column of $G^{*}$, called $\gamma^{\prime }$. Constraints C12 and C20 guaranteed that

(24) $$\gamma^{{}^{\prime }}\left(3j-2\right)+\gamma^{{}^{\prime }}\left(3j-1\right)+\gamma^{{}^{\prime }}\left(3j\right)=1,j=1,\ldots ,K,$$

where each element of $\gamma^{\prime }$ was a positive real number between 0 and 1. Therefore, we took $\gamma^{\prime }\left(j\right)$ as the probability of g(j)=1, for j=1,…,3K. We defined $\mathrm{pr}={\left[{pr}_{1}^{l},{pr}_{1}^{e},{pr}_{1}^{c},\ldots ,{pr}_{K}^{l},{pr}_{K}^{e},{pr}_{K}^{c}\right]}^{T}≜\gamma^{\prime }$, where each element of pr represented the probability of the corresponding entry of the offloading strategy being 1. Then, we generated a random column vector ξ with the size of *K* as a random variable of the stochastic mapping, which was based on the standard uniform distribution $\xi_{l}\;∼\;U\left(0,1\right)$. To recover the offloading strategy satisfying the constraint C1, we denoted a vector $v_{k}$ as the offloading decision of task *k*. The mapping relationship could be expressed as

(25) $$v_{k}=\left\{\begin{array}{cc} {}[1,0,0], & \xi \left(k\right)⩽pr_{k}^{l},\\ {}[0,1,0], & pr_{k}^{l}<\xi \left(k\right)⩽pr_{k}^{e},\\ {}[0,0,1], & \xi \left(k\right)>pr_{k}^{e}. \end{array}\right.$$

We generated a random sample $\xi^{\prime }={\left[v_{1},\ldots ,v_{K}\right]}^{T}$ by the mapping method Equation (25). However, $\xi^{\prime }$ was not always a feasible solution due to the delay constraint C13. Next, the ready time and the finish time of each task were computed through the random sample $\xi^{\prime }$, and ${FT}_{K}$ was further obtained. If ${FT}_{K}>T_{s}^{max}$, it indicated that the random sample $\xi^{\prime }$ did not meet the latency constraint and it would be discarded. Conversely, $\xi^{\prime }$ was a feasible solution for the optimization problem Equation (15), denote as $\hat{\xi }$.

To obtain a more accurate offloading strategy, we generated L random samples and obtained feasible solutions ${\hat{\xi }}^{\left(l\right)}$ by performing the above procedure. We let the subscript (l) denote the index of a random sample. We then chose among these feasible solutions the one that minimized the objective function of the optimization problem Equation (15) as the offloading strategy $\xi^{*}$. For the best offloading strategy, in practice, we compared $\xi^{*}$ with local computing only and cloud executing only solutions, and chose the solution with the minimum energy cost as the final offloading strategy $\gamma^{*}$. The details of the ECTCO algorithm are described in Algorithm 1.

Notice that the SDP problem in Step 2 of Algorithm 1 could be solved readily within a precision ϵ by using the interior point method in $O(\sqrt{K}\log\left(1/\epsilon \right))$ iterations, where the computational complexity per iteration is $O(K^{3})$ [30]. Thus, the computational complexity of ECTCO is $O(K^{3.5}\log\left(1/\epsilon \right)+LK)$.

**Algorithm 1** Proposed ECTCO algorithm**Input:***K*,*B*,$\sigma^{2}$,$T_{s}^{max}$,$G_{s}$. $\omega_{k}$,$d_{k}$,$H_{k}$,$p_{k}^{tra}$,$p_{k}^{cir}$,$f_{k}^{l}$,$f_{k}^{e}$,$f_{k}^{c}$,$R_{k}^{EC}$,∀k∈K. **Output:**$\gamma^{*}$. 1:**Initialize:** compute P(k) by $G_{s}$ and initialize all the matrices in Equation (22).2:solve the SDP problem Equation (22) without the rank-1 constraint to get its optimal solution $G^{*}$.3:extract the first 3K elements from the last column of $G^{*}$ as $\gamma^{\prime }$.4:**if**$\mathrm{rank}(G^{*})=1$**then**5:    construct $\gamma^{*}$ from $\gamma^{\prime }$.6:**else**7:    **for** l=1 to *L* **do**8:        generate random column vector $\xi_{l}\;∼\;U\left(0,1\right)$.9:        obtain random sample $\xi_{l}^{\prime }$ by offloading probability based stochastic mapping method according to Equation (25).10:        k=1;11:        **repeat**12:           **if** P(k)=⌀ **then**13:               ${RT}_{k}=0$;14:           **else**15:               compute $RT_{k}=\max_{j\in P(k)}{FT}_{j}$;16:           **end if**17:           compute $FT_{k}$ by $\xi_{l}^{\prime }$ and Equation (8);18:           k=k+1;19:        **until** k=K+120:        **if** $FT_{K}>T_{s}^{max}$ **then**21:           discard the random sample $\xi_{l}^{\prime }$.22:        **else**23:           $\xi_{l}^{\prime }$ as feasible solution ${\hat{\xi }}^{\left(l\right)}$,compute the energy cost by ${\hat{\xi }}^{\left(l\right)}$ and Equation (14) as $E^{\left(l\right)}$.24:        **end if**25:    **end for**26:    choose the solution among ${\hat{\xi }}^{\left(1\right)},\ldots ,{\hat{\xi }}^{\left(L\right)}$ that yields the minimum energy cost: $\xi^{*}=\underset{{\hat{\xi }}^{\left(l\right)}}{\mathrm{argmin}}\;E^{\left(l\right)}$.27:    compare $\xi^{*}$ with local computing only and cloud computing only solutions, update the one that yields the minimum energy cost as $\gamma^{*}$.28:**end if**

## 5. Simulation Results

In this section, extensive simulations are conducted to evaluate the performance of the ECTCO algorithm. The simulation settings will be first presented, followed by simulation results that verify the effectiveness of the proposed ECTCO algorithm in minimizing energy cost.

### 5.1. Simulation Settings

We simulated a cloud-assisted edge computing system and realized the proposed ECTCO algorithm in the Matlab environment. The system consisted of multiple IoT sensors, an edge server, and a cloud server. We randomly generated task graphs, i.e., directed acyclic graph (DAG), with *K* computing tasks and an ending node. Simulation results in this section are based on an average over 1000 random simulations. Moreover, our simulations were performed on a PC with Intel Core i5-7400 processor @ 3.0 GHz CPU and 8 GB of RAM. The main simulation parameters referred to by some previous works [22,31], unless mentioned otherwise, are listed in Table 2.

To evaluate the performance of the proposed ECTCO algorithm, we also simulated the following four algorithms for comparison.

- Offloading nothing algorithm (OLNA): All computing tasks were executed on their own sensors.
- Cloud-first offloading algorithm (CFOA): We offloaded all computing tasks to the cloud server.
- Execution-energy greedy offloading strategy (EGOS): For each computing task on IoT sensors, it was greedily offloaded to the computation node that resulted in the minimizing executing energy consumption. The computation node included local sensor, edge server, and cloud server.
- Joint resource allocation and offloading decision (JRAO) [42]: Jointly optimized the allocation of communication resource and the offloading decisions of IoT sensors. All computing tasks could be performed on their own sensors or offloaded to the edge server without consideration of the inter-task dependency relationship.

### 5.2. Performance of the ECTCO Algorithm

**Figure 3:**
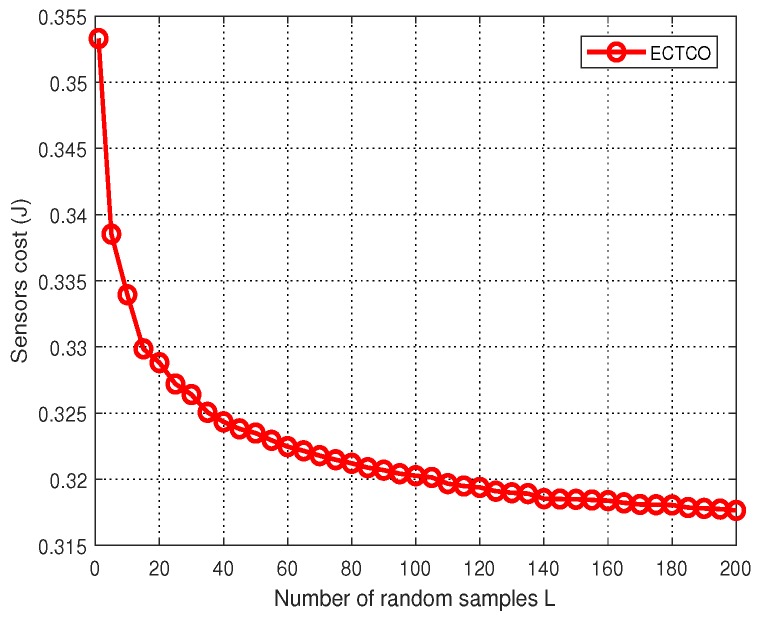
Sensors cost versus the number of random samples *L*.

Figure 3 plots the energy consumption of IoT sensors versus the number of random samples. We observe that as the value of *L* increased, the total energy consumption of IoT sensors decreased gradually. This was because the ECTCO algorithm obtained the offloading strategy by generating random samples based on the offloading probability. The larger the number of random samples were, the lower energy consumption of IoT sensors was. In addition, the sensors cost dropped sharply at the beginning, while slowed down with increasing *L*. It can be seen from Figure 3 that the rate of decrease in the sensors cost decelerated considerably about after L=100, which meant that beyond this point we had to use a larger *L* to achieve a marginal performance gain. For example, to decrease the sensors cost from 0.323 to 0.320, *L* needed to increase from 50 to 100; Meanwhile, to drop the sensors cost from 0.320 to 0.317, *L* needed to increase from 100 to over 200. Based on such trade-offs, it was reasonable to set L=100, which achieved better performance without too high computational complexity. Thus, we took L=100 as the default numbers of random samples in rest simulations.

**Figure 4:**
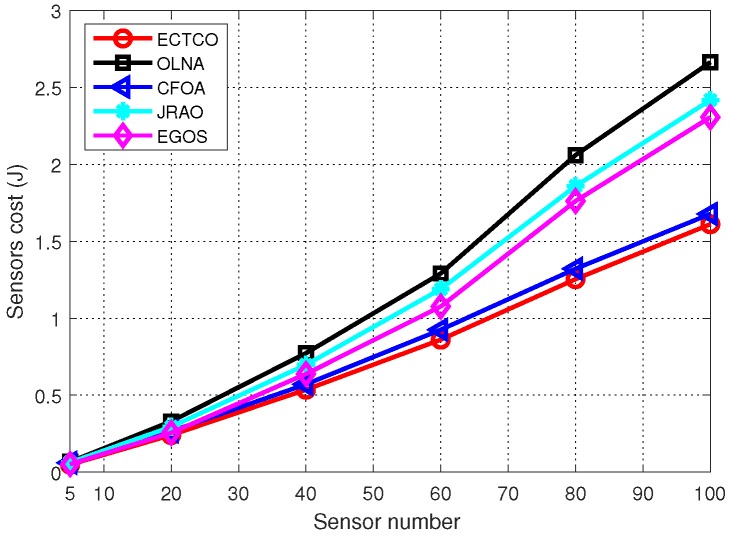
Sensors cost of different algorithms.

Figure 4 and Table 3 present the sensors cost under different algorithms, with 95% confidence interval (CI). The number of tasks *K* increased from 5 to 100 with the number of IoT sensors. Obviously, the sensors cost of all algorithms increased as the number of sensors grew. Furthermore, compared with the other four methods, the ECTCO algorithm reduced the sensors cost effectively. When the sensor number was small, the difference between the algorithms was not significant. As sensor number increased, the proposed ECTCO algorithm outperformed the other four methods. For example, when the sensor number was 60, the ECTCO algorithm could reduce 33.46%, 6.59%, 27.19% and 19.68% of the sensors cost in comparison to the schemes of OLNA, CFOA, JRAO and EGOS, respectively. It is worth noting that the JRAO algorithm could not obtain energy efficient offloading decisions due to the lack of consideration of inter-task dependency.

In addition, for the ECTCO and the EGOS algorithms, they could achieve similar energy consumption when the sensor number was small. However, as the sensor number grew, the energy consumption of EGOS increased rapidly. This was because EGOS is greedy for executing energy consumption. In more detail, according to Equation (3), the energy consumption of local computing depended on the computation capability of CPU. Thus, EGOS could select a sensor with low computation ability to perform a computing task. When the sensor number was small, the sensors cost was mainly composed of the executing energy consumption. In this situation, the EGOS had a satisfying performance with executing some tasks locally. As the sensor number increased, the waiting energy consumption could not be ignored due to the inter-task dependency relationship. The low computation ability of the sensor performing computing task resulted in a longer task completion time, which directly increased the waiting energy consumption of other sensors in this circumstance. Therefore, the EGOS consumed less energy with the growth of the sensor number while the ECTCO could obtain good trade-offs between the waiting energy consumption and the executing energy consumption to achieve a higher energy efficiency performance.

### 5.3. Impact of Different Parameters and Dependency Relationships

**Figure 5:**
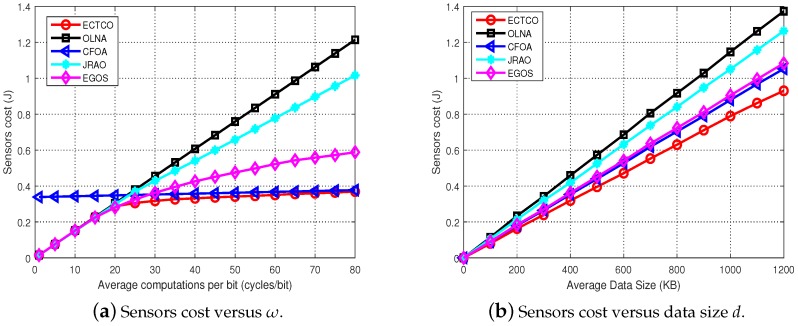
Impact of different parameters.

This part evaluates the effect of different parameters and dependency relationships on energy consumption of sensors. The influence of the average amount of computations per bit ω on different algorithms is illustrated as Figure 5a and Table 4, with 95% CI. We observe that as ω grew, the sensors cost performed by all algorithms tended to increase. For CFOA, the sensors cost increased slightly with growing ω. This was because all computing tasks were offloaded to the cloud server by CFOA and the cloud server had powerful computation resources. Meanwhile, when ω was less than 15, tasks could obtain the minimum energy consumption in local computing. Since the low computational complexity of the task, the computation cost of the tasks with local computing was less than communication cost of offloading tasks. These tasks could be viewed as communication-intensive tasks. Relatively, tasks offloading could obtain better energy efficiency with increasing ω, and the tasks could be considered as computation-intensive tasks. In addition, compared with the other four algorithms, the ECTCO algorithm could effectively obtain the lowest sensors cost under different ω, indicating that ECTCO could adaptively adjust the offloading strategy so as to efficiently achieve less energy consumption.

Figure 5b and Table 5 present the effect of average data size *d* on different algorithms, simulation results with 95% CI. Obviously, the sensors cost increased approximately linearly with the growing of *d*. The rate of increase in the sensors cost performed by the ECTCO was the slowest in comparison to the other four algorithms. This indicated that the proposed ECTCO algorithm performed better in energy consumption reduction under different average data sizes.

To evaluate the adaptability of the ECTCO algorithm, we analyzed the performance of the five schemes on the service completion time and energy consumption under different kinds of task dependency relationships. More specifically, we generated three kinds of task dependency relationships, i.e., fully sequential dependency, fully parallel dependency and arbitrary dependency [43]. Notice that the fully parallel dependency meant tasks could be executed in parallel except for the task in the ending node. Unless otherwise stated, the number of tasks K=25.

**Figure 6:**
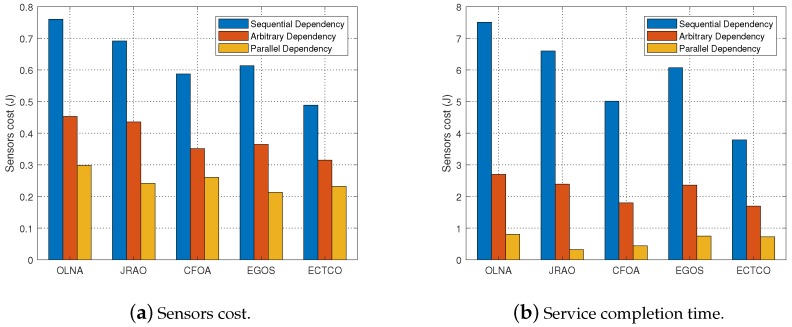
Impact of different dependency relationships.

Figure 6a and Table 6 show the total energy consumption of the five algorithms on different task dependency relationships, with 95% CI. We can find that our proposed ECTCO algorithm consumed the minimum energy in the fully sequential dependency and the arbitrary dependency compared with other algorithms. For the fully parallel dependency, the sensors cost performed by the ECTCO was close to the EGOS. This was because the waiting energy consumption was almost negligible under the fully parallel dependency. In this situation, the EGOS could always find an offloading strategy that minimized energy consumption. However, the waiting energy consumption had a significant impact on total energy consumption in the fully sequential dependency while the EGOS did not consider it, resulting in a large energy consumption under this circumstance. On the contrary, the ECTCO conserved energy effectively under different task dependency relationships.

In Figure 6b, we compared the service completion time of our proposed ECTCO algorithm with the other four algorithms under different task dependency relationships. It could be observed that different task dependencies had an impact on service completion time. For the same scheme, the fully sequential dependency required the longest service completion time while the fully parallel dependency was the shortest. Furthermore, we can also observe that only the ECTCO algorithm could meet the time constraint $T_{s}^{max}=4$ under different dependencies. The other three algorithms could not satisfy it in the fully sequential dependency. Therefore, the ECTCO algorithm could effectively reduce the sensors cost under different task dependencies while meeting the constraint of service completion time, demonstrating the adaptability of the ECTCO algorithm.

## 6. Conclusions and Future Work

In this paper, we investigate an energy conservation problem of IoT sensors in cloud-assisted edge computing framework by optimization of the computation offloading strategy. The energy conservation problem was formulated as an energy consumption minimization problem while meeting the constraints of inter-task dependency relationships and service completion time. To solve the NP-hard problem, we proposed the ECTCO algorithm, employing the SDR approach and the probability-based stochastic mapping method to obtain the computation offloading strategy.

In the simulation section, we evaluated the performance of the proposed ECTCO algorithm by comparing it with existing algorithms. Simulation results demonstrated that in the inter-task dependency scenario, the proposed algorithm could balance the tradeoff between computation and communication overhead, and outperform the other four algorithms in computation offloading in terms of energy consumption. In addition, we studied the impact of different system parameters and dependencies. Performance evaluations showed that the proposed algorithm could effectively reduce the sensors cost under different system parameters and dependencies. These simulation results verified the effectiveness and adaptability of the ECTCO algorithm.

In future work, we plan to deploy the proposed framework to real-world IoT scenarios so as to further conduct practical evaluations of the proposed algorithm. We also expect to explore mobility management and the offloading problem of tasks for sensors with inter-task dependency in a dynamic moving environment.

## Figures and Tables

**Table 1:** Model terminology.

Notation	Definition
$E_{k}^{loc},E_{k}^{edge},E_{k}^{cloud}$	Energy consumption for task *k* in local/edge/cloud computing
$E_{k}^{wait},E_{k}^{exec}$	Waiting/executing energy consumption for task *k*
$T_{k}^{loc},T_{k}^{edge},T_{k}^{cloud}$	Latency for task *k* in local/edge/cloud computing
$f_{k}^{l},f_{k}^{e},f_{k}^{c}$	The CPU cycles frequency of local/edge/cloud allocated to task *k*
$p_{k}^{tra},p_{k}^{cir}$	Idle circuit/transmission power of task *k*
$x_{k},y_{k},z_{k}$	Offloading strategies of task *k*
γ	The set of offloading strategy of all tasks
K,K	The set/number of computing task
$T_{s}^{max}$	Completion deadline for service *S*
$\omega_{k}$	CPU cycles spent for each bit in task *k*
$d_{k}$	Data size of task *k*
$C_{k}$	Size of CPU cycles amount required to complete task *k*
$r_{k}$	The transmission rate for task *k* between the sensor and the edge
*B*	The channel bandwidth between sensors and the edge
$H_{k}$	The channel gains for task *k* between the sensor and the edge
$\sigma^{2}$	The variance of complex white Gaussian channel noise
$R_{k}^{EC}$	The rate for task *k* between the edge and the cloud in wired link
*L*	Number of random samples

**Table 2:** Default parameters setup.

Parameters	Value
*K*	25
*B*	5 MHz
$\sigma^{2}$	$10^{-9}$ W
$H_{k}$	$10^{-6}$
$d_{k}$	300–500 KB uniformly
$\omega_{k}$	30 cycles/bit
$T_{s}^{max}$	4 s
$f_{k}^{l}$	0.1–0.5 G cycles/s uniformly
$f_{k}^{e}$	2 G cycles/s
$f_{k}^{c}$	4 G cycles/s
κ	$10^{-27}$
$p_{k}^{tra}$	0.1 W
$p_{k}^{cir}$	0.001–0.01 W uniformly
$R^{EC}$	5 MB/s

**Table 3:** Sensors cost versus sensor number.

Sensor Number	Sensors Cost (J)				
	ECTCO (95% CI)	OLNA (95% CI)	CFOA (95% CI)	JRAO (95% CI)	EGOS (95% CI)
5	0.048 (0.046, 0.050)	0.064 (0.060, 0.068)	0.059 (0.058, 0.061)	0.062 (0.060, 0.065)	0.050 (0.048, 0.053)
20	0.236 (0.233, 0.240)	0.320 (0.310, 0.329)	0.262 (0.259, 0.265)	0.297 (0.290, 0.303)	0.251 (0.245, 0.257)
40	0.533 (0.527, 0.538)	0.760 (0.742, 0.778)	0.567 (0.562, 0.572)	0.701 (0.687, 0.716)	0.634 (0.619, 0.649)
60	0.865 (0.856, 0.873)	1.300 (1.276, 1.325)	0.926 (0.916, 0.935)	1.188 (1.162, 1.214)	1.077 (1.052, 1.101)
80	1.247 (1.236, 1.257)	2.044 (2.009, 2.078)	1.317 (1.305, 1.328)	1.859 (1.822, 1.897)	1.761 (1.723, 1.798)
100	1.605 (1.594, 1.617)	2.634 (2.591, 2.677)	1.671 (1.659, 1.683)	2.424 (2.377, 2.471)	2.298 (2.254, 2.343)

**Table 4:** Sensors cost versus ω.

ω (cycles/bit)	Sensors Cost (J)				
	ECTCO (95% CI)	OLNA (95% CI)	CFOA (95% CI)	JRAO (95% CI)	EGOS (95% CI)
10	0.150 (0.148, 0.153)	0.150 (0.148, 0.153)	0.341 (0.339, 0.344)	0.151 (0.148, 0.154)	0.150 (0.148, 0.153)
20	0.284 (0.280, 0.287)	0.303 (0.297, 0.308)	0.347 (0.344, 0.351)	0.300 (0.295, 0.305)	0.280 (0.274, 0.285)
30	0.315 (0.312, 0.318)	0.453 (0.445, 0.461)	0.352 (0.349, 0.355)	0.418 (0.411, 0.426)	0.365 (0.357, 0.372)
40	0.331 (0.328, 0.334)	0.603 (0.592, 0.615)	0.358 (0.355, 0.362)	0.547 (0.539, 0.555)	0.430 (0.420, 0.440)
50	0.343 (0.339, 0.346)	0.762 (0.750, 0.775)	0.365 (0.362, 0.368)	0.665 (0.653, 0.677)	0.489 (0.475, 0.503)
60	0.351 (0.348, 0.355)	0.904 (0.888, 0.921)	0.368 (0.364, 0.371)	0.793 (0.779, 0.808)	0.528 (0.511, 0.545)
70	0.363 (0.360, 0.366)	1.064 (1.046, 1.081)	0.375 (0.372, 0.378)	0.911 (0.895, 0.927)	0.567 (0.544, 0.589)
80	0.370 (0.366, 0.373)	1.200 (1.177, 1.223)	0.378 (0.375, 0.382)	1.031 (1.014, 1.048)	0.588 (0.565, 0.611)

**Table 5:** Sensors cost versus data size *d*.

Average Data Size (KB)	Sensors Cost (J)				
	ECTCO (95% CI)	OLNA (95% CI)	CFOA (95% CI)	JRAO (95% CI)	EGOS (95% CI)
200	0.160 (0.158, 0.162)	0.230 (0.226, 0.235)	0.177 (0.175, 0.180)	0.218 (0.213, 0.222)	0.182 (0.178, 0.187)
400	0.315 (0.312, 0.318)	0.453 (0.445, 0.461)	0.352 (0.349, 0.355)	0.418 (0.411, 0.426)	0.365 (0.357, 0.372)
600	0.472 (0.467, 0.476)	0.682 (0.667, 0.696)	0.527 (0.523, 0.531)	0.632 (0.622, 0.643)	0.543 (0.530, 0.555)
800	0.628 (0.623, 0.633)	0.907 (0.893, 0.921)	0.703 (0.698 0.708)	0.839 (0.827, 0.852)	0.727 (0.714, 0.739)
1000	0.784 (0.778, 0.789)	1.131 (1.113, 1.149)	0.877 (0.871, 0.883)	1.050 (1.033, 1.068)	0.905 (0.888, 0.922)
1200	0.933 (0.927, 0.939)	1.379 (1.356, 1.402)	1.057 (1.050, 1.064)	1.262 (1.242, 1.283)	1.111 (1.089, 1.133)

**Table 6:** Sensors cost versus dependency relationships.

Dependency	Sensors Cost (J)				
	ECTCO (95% CI)	OLNA (95% CI)	CFOA (95% CI)	JRAO (95% CI)	EGOS (95% CI)
Sequential	0.492 (0.486, 0.499)	0.763 (0.751, 0.775)	0.592 (0.584, 0.601)	0.693 (0.684, 0.702)	0.613 (0.601, 0.625)
Arbitrary	0.315 (0.312, 0.318)	0.453 (0.445, 0.461)	0.352 (0.349, 0.355)	0.418 (0.411, 0.426)	0.365 (0.357, 0.372)
Parallel	0.236 (0.234, 0.239)	0.301 (0.295, 0.307)	0.262 (0.261, 0.264)	0.241 (0.240, 0.243)	0.217 (0.215, 0.220)
